# Carbon Information Disclosure, Marketization, and Cost of Equity Financing

**DOI:** 10.3390/ijerph16010150

**Published:** 2019-01-08

**Authors:** Li Li, Quanqi Liu, Jun Wang, Xuefei Hong

**Affiliations:** 1 School of Economics and Management, Harbin Institute of Technology, Shenzhen 518055, China; ximlli@126.com (L.L.); wangjunhnust@126.com (J.W.); hxfhit2006@163.com (X.H.); 2 School of International Economics and Trade, Jiangxi University of Finance and Economics, Nanchang 330013, China; 3 School of Business, Hunan University of Science and Technology, Xiangtan 411201, China

**Keywords:** carbon information disclosure, marketization, cost of equity financing

## Abstract

Using listed enterprises in China’s heavy pollution industry from 2009 to 2013, this study tests the relationship between marketization degree, carbon information disclosure, and the cost of equity financing. The results show that, regardless of marketization degree, the overall level of carbon information disclosure of listed enterprises in China’s heavy pollution industry is low. The content of carbon information disclosure is mainly non-financial carbon information, and the financial carbon information disclosure is very low. The cost of equity financing is different in areas with different marketization degrees, specifically speaking, the cost of equity financing is lower in regions with a high marketization degree than that of a low marketization degree. Carbon information disclosure, non-financial carbon information disclosure, and financial carbon information disclosure are negatively correlated with the cost of equity financing. The marketization degree has strengthened the negative correlation between carbon information disclosure, non-financial carbon information disclosure, financial carbon information disclosure, and the cost of equity financing, respectively.

## 1. Introduction

In order to ensure the implementation of the carbon emissions trading system countrywide in 2017, the National Development and Reform Commission issued the “Notice on the Key Work of Starting the National Carbon Emission Trading Market” [1]. Carbon emission reduction will generate a direct cost to enterprises, which will increase the financial pressure on enterprises, so enterprises need to find a way to reduce cost. In fact, carbon information disclosure can enhance the transparency of information and reduce information asymmetry, which is also an effective way to reduce the financial risk of investors [2,3]. At the same time, in the capital market, the improvement of the quality of enterprise carbon information disclosure will promote the reduction of enterprise capital cost [4,5]. Due to the different preferences of investors, as an ecological benefit management strategy of enterprises, non-financial information disclosure will affect their capital costs [6]. China’s capital market is representative of the emerging capital market, where the relationship between enterprise information disclosure and capital cost is similar to that of a mature capital market; that is, enterprises can reduce the cost of capital by disclosing relevant information [7]. With the continuous development of China’s market economy, marketization degree shows different levels in different regions. To some extent, the regional differences will affect the decision behavior of enterprises in these areas. The higher the marketization degree, the greater the possibility of enterprises disclosing high-quality internal control information [8]. The high marketization degree means less government intervention, lower political relevance and less political connection will increase the predictability of enterprises and reduce the cost of capital [9]. The legal system in this area is relatively perfect and standardized, and as people can handle affairs according to the law, the capital cost is significantly lower [10].

The existing research mainly has the following characteristics: (1) In the terms of data sources, the extant research is mainly based on CDP (Carbon Disclosure Project), but does its index system framework conform to the behavior style of Chinese enterprises? Is it really accepted by Chinese companies? In fact, except for a few large enterprises participating in this project, most Chinese enterprises are not very enthusiastic in this matter, which will inevitably affect the representativeness of the relevant domestic research results. According to the results of CDP China Report—in the CDP China 100 survey, the number of enterprises responding to the questionnaire and providing information from 2011 to 2014 was 21, 23, 32 and 45, respectively—it can be seen that less than 50% of the 100 enterprises invited are willing to participate in this survey project, and as far as the CDP China Report of 2011 is concerned, the banking industry is more motivated to fill in the questionnaire, accounting for 36% of the enterprises that fill in the questionnaire [11]; (2) as for the marketization degree, the existing research mainly focuses on the environmental information disclosure, and the literature is very rich. However, there are few intensive studies on the sub-field of environmental information disclosure, carbon information disclosure, and a lack of literature on the relationship between carbon information disclosure and cost of equity financing from the perspective of marketization degree.

China has already put forward a policy of building an ecological civilization, and low-carbon development is also an inevitable choice, which will play an important role in alleviating environmental pressure and reversing the situation of continued deterioration of the ecological environment. Carbon reduction positively and significantly influences corporate sustainable development [12]. Carbon information disclosure is an important issue in the development of low-carbon, which has widely concerned stakeholders. With the continuous development of the market economy in China, the marketization degree will be further improved in general, but the marketization degree in various regions of the country is still uneven. Regions with different marketization degree have different political relevance, which will inevitably affect the decision of enterprises of different nature. Different companies may also strategically disclose different types of carbon information to attract the attention of investors. Based on the current background of China, this paper chooses the listed companies of heavy pollution industries in China from 2009 to 2013 as samples, and empirically tests the relationship between carbon information disclosure and cost of equity financing from the perspective of marketization degree, and further tests them according to the different types of carbon information.

The main contributions of this paper are as follows: (1) Deeply studying the field of environmental information disclosure, namely carbon information disclosure, evaluating the level of carbon information disclosure by building an index system, classifying carbon information into non-financial carbon information and financial carbon information, and examining the influence of different types of carbon information on capital cost; (2) taking the marketization degree as the breakthrough point, this paper analyses the influence of marketization degree on the relationship between carbon information disclosure and cost of equity financing, and broadens the research perspective of carbon information disclosure.

## 2. Theoretical Analysis and Hypothesis

**Figure 1:**
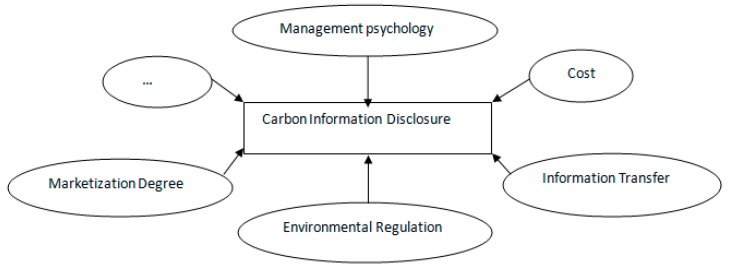
Influencing factors of carbon information disclosure.

In the current “information explosion” society, carbon information disclosure will be influenced by many factors [13,14], such as management psychological factor [15], information transfer factor, cost factor, environmental regulation factor [16], marketization degree factor, and more (Figure 1). To some extent, these factors affect the level of the developing trend of carbon information disclosure.

According to the theory of asymmetric information, in the market economy activities, different people have different information on the activities. Obviously, companies’ managers have more information about the company than the potential investors. From the stakeholder—agent theory and information asymmetry theory, as an important stakeholder of an enterprise, investors are prone to making an inaccurate investment decision because of their information disadvantage, which leads to adverse selection in the market. To a certain extent, carbon information disclosure can reduce information asymmetry. Furthermore, Kolk et al. found that CDP (Carbon Disclosure Project) has been successfully using institutional investors to urge firms to disclose extensive information about their climate change activities [17]. Signaling theory holds that in order to prevent investors from adverse selection, enterprises are willing to disclose more information about enterprises, release signals to investors, arouse investors’ attention, minimize the degree of information asymmetry, and enhance investors’ confidence. Investors generally believe that information disclosure is a favorable signal [18]. Research by Kim and Lyon showed that institutional investor activism towards climate change can increase shareholder value when the external business environment becomes more climate conscious [19]. Enterprises reducing the degree of information asymmetry can promote the decline of cost of equity financing [20]. Most of the existing literature believes that there is a significant negative correlation between information disclosure and the cost of capital [21]. By using data from S and P 500 firms that participated in the Carbon Disclosure Project (CDP) in 2010, He et al. found that the cost of capital is negatively associated with carbon disclosure, which is consistent with voluntary disclosure theory [22]. Investors’ expected risk and stock liquidity affect the relationship between information disclosure and the cost of equity financing; the higher the quality of information disclosure, the higher the liquidity of stock and the smaller the expected risk, the lower the cost of equity financing [4,7,23]. This relationship applies to both non-financial and financial information [21], but it should pay more attention to financial information disclosure [5,6]. The financial information disclosure can strengthen the negative relationship between carbon information disclosure and the cost of equity financing [24]. There is a study that indicates that non-financial information has little impact on investment decisions [25]. Some research results show that information asymmetry is not the main factor affecting the cost of equity financing [26]. Most investors are inclined to invest in enterprises with transparent information and less expected risk. [27]. Herold found that overall shifts to more transparent corporate carbon disclosure strategies correlated with an increase of applied carbon management practices in both internal and external actions [28]. Qian et al. pointed out that EMA (environmental management accounting) application has a significantly positive impact on both corporate carbon management and disclosure quality [29]. Enterprises should consider disclosing more carbon information, enhancing the communication effect with investors, reducing the uncertainty of investment decisions and avoiding adverse selection of investors. Lee et al. suggested that a firm can mitigate negative market shock from its carbon disclosure by releasing its carbon news periodically through the media in advance of its carbon disclosure [30]. In general, information disclosure has the function of reducing the cost of equity financing, therefore, the following assumptions are proposed:

**Hypothesis** **1** **(H1).** *Carbon information disclosure is negatively correlated with the cost of equity financing.*

**Hypothesis** **2** **(H2).** *Non-financial carbon information disclosure is negatively correlated with the cost of equity financing.*

**Hypothesis** **3** **(H3).** *Financial carbon information disclosure is negatively correlated with the cost of equity financing.*

**Figure 2:**
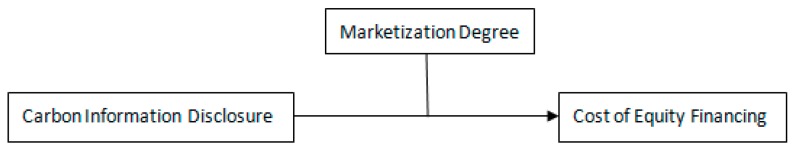
The moderating effect of marketization degree.

The marketization degree includes the level of economic development, the perfection of the legal system construction, and even the public’s awareness of environmental protection. The imbalance of regional development is a common phenomenon, so marketization degree is different in various regions. While the impact of marketization degree on carbon information disclosure can’t be ignored, the carbon information disclosure of enterprises may also be different. Marketization degree has a moderating effect on the relationship between carbon information disclosure and the cost of equity financing (Figure 2).

The system theory holds that integrity, relevance and dynamics are a common feature of all systems. Enterprises are an important part of the market economy system, and the economic operation of each enterprise will be constrained by the political, legal, market, and other external conditions and systems. Herold and Lee used data from Bloomberg ESG (Environmental Social Governance) and the Carbon Disclosure Project (CDP) reports to test the influence of internal and external pressures on carbon management practices and disclosure strategies, and found that these companies either are engaged in both internal and external practices or in neither. In fact, the key internal drivers are the companies’ policies and procedures, while key external drivers include high engagement with policy makers and NGOs [31]. The theory of new institutional economics emphasizes that institution and market mechanisms play an equally important role in the allocation of resources and economic decisions. Luo et al. found that the carbon disclosure propensity is correlated in the right direction with resource availability proxies; this relationship is stronger in developing nations [32]. Marketization degree reflects the role of market mechanism in the allocation of resources, but also shows the perfection of the system. The marketization degree plays an important role in China’s economic growth [33]. Marketization reform promotes the efficiency of resource allocation and among them, the contribution of marketization process is remarkable. However, there is still a long way to go for China’s marketization transformation, as the sustainable development of China’s economy depends on the promotion of the marketization process [34]. The marketization process will inevitably bring about changes in the institutional environment [35]. Under uncertain conditions, enterprises need to keep an eye on the process of institutional change and observe the impact of institutional change on transaction costs [36]. In areas with a high marketization degree, the development level of the markets is higher and the contract economy is more mature, which will strengthen the motivation of information disclosure in transactions of the capital market [37]. Marketization degree affects the transparency of information. In regions with higher marketization degree, the transparency of an enterprises’ information disclosure is higher [38]. For investors, a high marketization degree can reduce the adverse impact of information asymmetry [39]. Under certain conditions, enterprises with a high marketization degree are more inclined to fulfill more social responsibilities [40,41]. The capital cost of enterprises varies with the marketization degree in different regions [42]. In areas with a lower marketization degree, higher political relevance reduces the allocation efficiency of capital market, which leads to an increase of the capital costs; in areas with a higher marketization degree, enterprises are less interfered with the government, and the marketization degree can reduce corruption, which is conducive to reducing non-operating costs. Therefore, the cost of equity capital of enterprises is also lower [43]. To a certain extent, marketization degree can strengthen the negative correlation between information disclosure and enterprise capital cost. To this end, this paper puts forward the following assumptions:

**Hypothesis** **4** **(H4).** *In areas with high marketization degree, the significant negative correlation between carbon information disclosure and the cost of equity financing will be strengthened.*

**Hypothesis** **5** **(H5).** *In areas with high marketization degree, the significant negative correlation between non-financial carbon information disclosure and the cost of equity financing will be strengthened.*

**Hypothesis** **6** **(H6).** *In the areas with high marketization degree, the significant negative correlation between financial carbon information disclosure and the cost of equity financing will be strengthened.*

## 3. Research Design

### 3.1. Sample Selection and Data Sources

Taking listed enterprises of China’s heavy pollution industry in Shanghai and Shenzhen Stock Exchanges as samples, this paper chooses social responsibility reports or sustainable development reports issued by the listed enterprises in heavy pollution industry from 2009 to 2013 as data sources of carbon information disclosure, excluding ST (listed enterprises that have suffered losses for two consecutive years and have been specially treated), * ST (listed enterprises have suffered losses for three consecutive years and have been warned of delisting risks), and data missing enterprises, with a total of 128 enterprises. Other data are from CSMAR database, GFP database, and Resset database.

### 3.2. Variables

#### 3.2.1. Carbon Information Disclosure and Classification

The carbon information disclosure index system was built based on the previous research [44]. CID (Carbon Information Disclosure) indicates the carbon information disclosure variable, using the content analysis method to evaluate carbon information disclosure of the sample enterprise. The specific evaluation items, standards, and instructions are as follows: low-carbon development strategy, establishment of a low-carbon management department, promotion of employees’ low-carbon awareness, and carbon emission reduction is recognized by the government and incorporating low-carbon development into performance appraisals equals 1 or 0; investment and achievements in low-carbon scientific research, exploitation and utilization of resources, and benefits of developing a low-carbon economy equals 2, 1, or 0; description of carbon emissions equals 3, 2, 1.5, 1 or 0.

In addition, referring to the practice of Ye et al., carbon information disclosure is classified into non-financial carbon information disclosure (CIDNF) and financial carbon information disclosure (CIDF) [24]. Non-financial carbon information disclosure’s items include low-carbon development strategy, the establishment of low-carbon management, and promotion of employees’ low-carbon awareness. Carbon emission reduction is recognized by the government, as is incorporating low-carbon development into performance appraisal. Financial carbon information disclosure’s items include carbon emissions, investment and achievements in low-carbon scientific research, exploitation and utilization of resources and benefits of developing a low-carbon economy. The calculation formula of carbon information disclosure is as follows [44]:

(1) $${\mathrm{CID}}_{i}=\frac{\sum {\mathrm{CIDP}}_{i}}{\mathrm{MCID}}$$

${\mathrm{CID}}_{i}$ refers to carbon information disclosure of enterprise *i*. $\sum {\mathrm{CIDP}}_{i}$ refers to the sum of all the enterprise’s disclosure item scores; MCID refers to the sum of the highest score of all the disclosure items and MCID is 14.

The calculation formula of non-financial carbon information disclosure is as follows:

(2) $${\mathrm{CIDNF}}_{i}=\frac{\sum {\mathrm{CIDNFP}}_{i}}{\mathrm{MCIDNF}}$$

${\mathrm{CIDNF}}_{i}$ refers to non-financial carbon information disclosure of enterprise *i.* $\sum {\mathrm{CIDNFP}}_{i}$ refers to the sum of all the enterprise’s disclosure item scores. MCIDNF refers to the sum of the highest score of all the disclosure items and MCIDNF is 8.

The calculation formula of financial carbon information disclosure is as follows:

(3) $${\mathrm{CIDF}}_{i}=\frac{\sum {\mathrm{CIDFP}}_{i}}{\mathrm{MCIDF}}$$

${\mathrm{CIDF}}_{i}$ refers to the financial carbon information disclosure of enterprise *i*. $\sum {\mathrm{CIDFP}}_{i}$ refers to the sum of all the enterprise’s disclosure item scores. MCIDNF refers to the sum of the highest score of all the disclosure items and MCIDF is 6.

#### 3.2.2. Marketization

Marketization degree of this paper mainly refers to data from Fan and Wang’s “China’s Marketization Index: Relative Process of Marketization in Various Regions Annual Report for 2011”, and combines with the practices of the existing literature [37,45]. The explanation of marketization degree is shown in Table 1 [37]. The specific method is to regard marketization degree as a dummy variable, and the marketization degree (MI) of Zhejiang, Shanghai, Jiangsu and Guangdong provinces and municipalities directly under the Central Government is taken as 1, which indicates that the marketization degree of these regions is high, while that of other regions is 0, indicating that the marketization degree is low. According to the above literature, the reason for this is that the four regions ranked as the top four for marketization degree from 2004 to 2009, and the data for the next few years are missing, so the dummy variable is used to measure it.

#### 3.2.3. Cost of Equity Financing

In this paper, we use the economic growth model to calculate the cost of equity financing, referring to the practice of Ye et al. [24]. This method takes into account the applicability and science of the method and the availability of the data. At the same time, in order to test the stability of the results, the PEG (price/earnings to growth) scale model is used to calculate the cost of equity financing and test results. The formula for calculating the cost of equity financing is as follows:

(4) $$\mathrm{CEF}=\frac{1}{2}\left[\left(\gamma -1\right)+\frac{\delta \times eps_{1}}{p_{0}}\right]+\sqrt{\frac{1}{4}{\left[\left(\gamma -1\right)+\frac{\delta \times eps_{1}}{p_{0}}\right]}^{2}+\frac{eps_{1}}{p_{0}}\left[\frac{eps_{2}-eps_{1}}{eps_{1}}-\left(\gamma -1\right)\right]}$$

Among them, the cost of equity financing is CEF. Long term earnings growth rate is γ−1; δ is the average dividend payout ratio over the past three years. $eps_{1}$ is forecast of earnings per share in the year *t* + 1; $eps_{2}$ is forecast of earnings per share in the year *t* + 2; $p_{0}$ is the closing price of the shares at the end of year *t* − 1.

### 3.3. Control Variables

The control variables include financial leverage (FL), enterprise growth (OIGR), enterprise scale (CS), the proportion of independent directors (PID), the book market ratio (BM), beta (β), and part-time positions (DP). All variables are specified and signed in Table 1.

### 3.4. Models

In order to verify the relationship between marketization degree, carbon information disclosure and the cost of equity financing, we constructed the models according to the research hypothesis. Specific models are as (5)–(10):

(5) $$CEF_{i,t}=a_{0}+a_{1}CID_{i,t-1}+a_{2}FL_{i,t}+a_{3}OIGR_{i,t}+a_{4}CS_{i,t}+a_{5}PID_{i,t}+a_{6}BM_{i,t}+a_{7}\beta_{i,t}+a_{8}DP_{i,t}+\lambda$$

(6) $$CEF_{i,t}=b_{0}+b_{1}CIDNF_{i,t-1}+b_{2}FL_{i,t}+b_{3}OIGR_{i,t}+b_{4}CS_{i,t}+b_{5}PID_{i,t}+b_{6}BM_{i,t}+b_{7}\beta_{i,t}+\;b_{8}DP_{i,t}+\lambda$$

(7) $$CEF_{i,t}=c_{0}+c_{1}CIDF_{i,t-1}+c_{2}FL_{i,t}+c_{3}OIGR_{i,t}+cCS_{i,t}+c_{5}PID_{i,t}+c_{6}BM_{i,t}+c_{7}\beta_{i,t}+\;c_{8}DP_{i,t}+\lambda$$

(8) $$CEF_{i,t}=d_{0}+d_{1}CID_{i,t-1}+d_{2}MI_{i,t}+d_{3}CID_{i,t-1}\times MI_{i,t}+d_{4}FL_{i,t}+d_{5}OIGR_{i,t}+d_{6}CS_{i,t}+\;d_{7}PID_{i,t}+d_{8}BM_{i,t}+d_{9}\beta_{i,t}+d_{10}DP_{i,t}+\lambda$$

(9) $$CEF_{i,t}=e_{0}+e_{1}CIDNF_{i,t-1}+e_{2}MI_{i,t}+e_{3}CIDNF_{i,t-1}\times MI_{i,t}+e_{4}FL_{i,t}+e_{5}OIGR_{i,t}+e_{6}CS_{i,t}+e_{7}PID_{i,t}+e_{8}BM_{i,t}+e_{9}\beta_{i,t}+e_{10}DP_{i,t}+\lambda$$

(10) $$CEF_{i,t}=f_{0}+f_{1}CIDF_{i,t-1}+f_{2}MI_{i,t}+f_{3}CIDF_{i,t-1}\times MI_{i,t}+f_{4}FL_{i,t}+f_{5}OIGR_{i,t}+f_{6}CS_{i,t}+\;f_{7}PID_{i,t}+f_{8}BM_{i,t}+f_{9}\beta_{i,t}+f_{10}DP_{i,t}+\lambda$$

Among them, in the model (8)–(10), $CID_{i,t-1}\times MI_{i,t},CIDNF_{i,t-1}\times MI_{i,t}\;\mathrm{and}\;CIDF_{i,t-1}\times MI_{i,t}$ is the multiplication of the marketization degree and carbon information disclosure, non-financial carbon information disclosure and financial information disclosure. In order to control the potential endogeneity of carbon information disclosure and cost of equity financing, referring to the practice of Li and Liu [45], this paper will use the lag phase of carbon information disclosure and other variables.

## 4. Empirical Results and Analysis

### 4.1. Descriptive Statistics

Table 2 shows the descriptive statistics of different types of carbon information disclosure, cost of equity financing and other variables under different marketization degree conditions. In the high marketization degree area, the mean of carbon information disclosure (CID), non-financial carbon information disclosure (CIDNF), and financial information disclosure (CIDF) are 0.294, 0.374, and 0.188, respectively, while the mean of cost of equity financing (CEF) is 0.189. In the lower marketization degree area, the mean of carbon information disclosure (CID), non-financial carbon information disclosure (CIDNF), and financial information disclosure (CIDF) is 0.308, 0.388, and 0.201, respectively, while the mean of cost of equity financing (CEF) is 0.209. The results show that both the marketization degree and carbon information disclosure of Chinese listed enterprise in heavy pollution industries are low. Most of the carbon information disclosure is non-financial carbon information. Financial information disclosure is very low, even lower than the mean of carbon information disclosure. Compared to the low marketization degree region, the cost of equity financing of the high marketization degree is lower, which means that the cost of equity financing is different in various regions as the different marketization degrees.

### 4.2. Multiple Regression Results

This paper uses White Test to test whether the model has heteroscedasticity. The results are shown in Table 3. The results show that the *F*-value is greater than the adjusted *R*^2^ value, indicating that the regression models (5)–(10) are not exist heteroscedastic. The VIF values of each model are significantly less than 2, showing that these models are not exist multicollinear. Therefore, these models can be used for multiple regression analysis to examine the relationship between the carbon information disclosure and the cost of equity financing under different marketization degree conditions.

#### 4.2.1. The Relationship between Cost of Equity Financing and Carbon Information Disclosure, Non-Financial Carbon Information Disclosure, and Financial Carbon Information Disclosure

In this paper, the multiple regression models (5)–(7) are used to test the impact of carbon information disclosure, non-financial carbon information disclosure, and financial carbon information disclosure on the cost of equity financing. The empirical results are shown in Table 4. In Table 4, column (1) shows that the regression coefficient between CID and CEF is −0.076, which is significantly negative correlation (*t* = −2.258). In column (2), the regression coefficient between CIDNF and CEF is −0.070, which is significantly negatively correlated (*t* = −2.390). In column (3), the regression coefficient between CIDF and CEF is −0.056, which is significantly negatively correlated (*t* = −1.919). This shows that the carbon information disclosure, non-financial carbon information disclosure, and financial carbon information disclosure and cost of equity financing are significantly negatively correlated, respectively. Hypothesis 1, Hypothesis 2, and Hypothesis 3 have passed empirical tests.

#### 4.2.2. The Influence of Marketization Degree on the Relationship between Carbon Information Disclosure and Cost of Equity Financing

In order to further test the effect of marketization degree on the relationship between carbon information disclosure and the cost of equity financing, this paper divided carbon information disclosure, non-financial carbon information disclosure, and financial carbon information disclosure into two groups in accordance with the marketization degree, and conducted an empirical test. The results are shown in Table 5. As can be seen from Table 5, after grouping by marketization degree, the cross-multiplier coefficients of the regression model passed the test at the 5% significance level. It shows that marketization degree has a significant moderating effect on the cost of equity financing. For groups with high marketization degree, the regression coefficients between carbon information disclosure, non-financial carbon information disclosure, and financial carbon information disclosure and cost of equity financing are −0.104 (*t* = −2.057), −0.072 (*t* = −1.979) and −0.099 (*t* = −1.985), respectively, indicating that they are significantly negatively related to cost of equity financing. In the low marketization degree group, the regression coefficients between carbon information disclosure, non-financial carbon information disclosure, and financial carbon information disclosure and cost of equity financing are −0.095 (*t* = −2.026), −0.105 (*t* = −2.588), and −0.091 (*t* = −2.314), respectively, which are also significantly negatively correlated.

To judge whether the adjustment function of marketization degree is strengthened or weakened, we can get it by calculating partial derivatives. When marketization degree is not considered, the regression coefficients between carbon information disclosure, non-financial carbon information disclosure, and financial carbon information disclosure and cost of equity financing are −0.076, −0.070, and −0.056, respectively (in Table 4). After considering marketization degree, their regression coefficients are −0.199, −0.177 and −0.190, respectively.

Obviously, considering the coefficient of the marketization degree, the absolute value is larger, indicating that the slope of the regression model has increased. It shows that the marketization degree has strengthened the role of this relationship. That is to say, marketization degree has a strong effect on the negative correlation between carbon information disclosure, non-financial carbon information disclosure, and financial carbon information disclosure and cost of equity financing, respectively. Therefore, Hypothesis 4, Hypothesis 5, and Hypothesis 6 are supported by empirical evidence.

#### 4.2.3. Robustness Analysis

In order to investigate the robustness of the regression results, this paper uses the PEG model to calculate the cost of equity financing, replacing the dependent variable (CEF). The model assumes that under the assumption of a zero divided payment, the difference between the stock price and the book value can represent the residual income:

(11) $$\mathrm{PEG}=\sqrt{\frac{eps_{2}-eps_{1}}{p_{0}}}$$

About the carbon information disclosure variables, using Shen and Feng’s approach [46], the number of rows of carbon information in corporate social responsibility reports and sustainable development report are divided by the number of rows in the annual reports, and the carbon information disclosure report (CIDR) is calculated after the standardized treatment. CIDR is an alternative variable of carbon information disclosure. We take CIDR with a marketization degree (MI) as CIDR × MI, and conduct multiple regression analysis with cost of equity financing (PEG) to test the robustness of the results, as shown in Table 6.

Table 6 shows that when the marketization degree is not grouped, the regression coefficient of CIDR is −0.076, and passes the test at the level of 5% significance. After grouping, the high marketization degree multiplication coefficient is −0.071, which is significant at the 5% level, and the regression coefficient of CIDR with a low marketization degree is −0.096, which also passes the test by the significance level of 5%. According to the former method, the absolute value of the coefficient (0.167) after grouping is greater than that before grouping (0.076). The slope increases, indicating that marketization degree will strengthen the negative correlation between carbon information disclosure and cost of equity financing. The test results are basically consistent with the above empirical results, which show that the above conclusions are robust and reliable.

## 5. Discussion

On the one hand, the results are consistent with the findings in previous literature [4,6,10,21]. On the other hand, these results are not only consistent with the results in previous studies [42,43], but also further show that marketization degree has a moderating effect. Little attention has been paid to the moderating effect of marketization degree on the relationship between carbon information disclosure and cost of equity financing in extant literature.

The current situation determines the difference of marketization degree in different regions in China. The objective environment will have different effects on enterprise carbon information disclosure decisions and even affect the cost of financing in the capital market. The results provide empirical support for the formulation and improvement of China’s carbon information disclosure policies and regulations. In addition, the results will enrich the relevant research literature.

The following policy implications are proposed. The conclusion shows that promoting marketization reform is an effective way to improve the carbon information disclosure. The perfection of a market mechanism system is an important aspect of marketization reform and construction. We should improve the transparency of information, reduce unnecessary intervention of government, and effectively stimulate the enthusiasm of enterprises for low-carbon development. Therefore, the government should continue to steadily promote the process of marketization reform from the macro perspective, providing a good external business environment for enterprises.

Although the viewpoint of this paper is clear, the arguments are sufficient, and the logic is clear, there are still some limitations. Firstly, this paper finds that carbon information disclosure can reduce the cost of equity financing, but does not propose specific incentives to promote disclosing more carbon information. This is not conducive to improving the enthusiasm of enterprises to disclose carbon information. We will consider building a systematic incentive mechanism to improve the quality of carbon information disclosure in the future research, especially the improvement of financial carbon information. Secondly, this paper empirically examines the moderating effect of marketization degree using virtual variables to measure marketization degree, lacking empirical evidence of the actual value of market index, which may affect the public’s cognition of the marketization degree in some areas. After the publication of the market index, we can try to use the actual value of market index to study related issues. Finally, this paper does not put forward detailed suggestions and countermeasures from the perspective of policies and regulations, which reduces the operability of research conclusions in practical application. This is also a direction for our future research.

## 6. Conclusions

On the basis of previous studies, this paper takes the listed companies in China’s heavy pollution industries from 2009 to 2013 as samples to test the relationship between marketization degree, carbon information disclosure and cost of equity financing. The results show that, regardless of the degree of marketization, the overall level of carbon information disclosure of listed companies in China’s heavy pollution industry is low. The carbon information disclosure is mainly non-financial carbon information, and the disclosure of financial carbon information is very low, even lower than the mean value of carbon information disclosure. The cost of equity financing is different in different regions with different marketization degrees, which is manifested in the lower cost of equity financing in regions with a higher marketization degree than in regions with a lower marketization degree. It is found that carbon information disclosure, non-financial carbon information disclosure, and financial carbon information disclosure are significantly negatively correlated with the cost of equity financing, respectively. Furthermore, by testing the influence of marketization degree on the relationship between carbon information disclosure, non-financial carbon information disclosure, and financial carbon information disclosure and cost of equity financing, it is found that the marketization degree has the effect of strengthening the negative correlation between carbon information disclosure, non-financial carbon information disclosure, and financial carbon information disclosure and cost of equity financing respectively. 

## Figures and Tables

**Table 1:** Variables specification and description.

Variables	Symbol	Variable Description
Carbon Information Disclosure	CID	The general situation of carbon information disclosure
Non-Financial Carbon Information Disclosure	CIDNF	The situation of non-financial carbon information disclosure
Financial Information Disclosure	CIDF	The situation of financial carbon information disclosure
Marketization Degree	MI	High marketization degree equals 1; or else to be 0.
Cost of Equity Financing	CEF	Calculated using OJN model
Financial Leverage	FL	Enterprise asset liability ratio
Enterprise Growth	OIGR	Operating income growth ratio
Enterprise Scale	CS	The natural logarithm of the total assets of an enterprise at the end of the year
Proportion of Independent Directors	PID	The share of the number of independent directors in the board of directors
Book-to-market	BM	Net assets per share divided by share price
Beta	β	Beta coefficient
Part-time Position	DP	Chairman concurrently serves as general manager equals 1, or else is 0

**Table 2:** Descriptive statistics of different types of carbon information disclosure and other variables under different marketization degree conditions.

Group	High Marketization Degree (MI = 1, *N* = 160)			Low Marketization Degree (MI = 0, *N* = 480)		
Variables	Mean	Median	Standard Deviation	Mean	Median	Standard Deviation
CID	0.294	0.286	0.188	0.308	0.286	0.191
CIDNF	0.374	0.375	0.215	0.388	0.375	0.216
CIDF	0.188	0.167	0.204	0.201	0.167	0.221
CEF	0.189	0.155	0.128	0.209	0.154	0.184
FL	0.496	0.485	0.163	0.547	0.584	0.211
OIGR	0.134	0.117	0.213	0.141	0.116	0.252
CS	23.169	23.150	1.146	23.634	23.575	1.773
PID	0.352	0.333	0.099	0.350	0.333	0.105
BM	0.508	0.459	0.266	0.588	0.483	0.448
β	1.069	1.068	0.264	1.111	1.111	0.293
DP	0.231	0.000	0.423	0.090	0.000	0.286

**Table 3:** Regression analysis of marketization degree, carbon information disclosure and cost of equity financing.

Variables	Model (5)	Model (6)	Model (7)	Model (8)	Model (9)	Model (10)
CID	−0.105 *** (−3.060)					
CIDNF		−0.098 *** (−3.437)				
CIDF			−0.060 * (−1.875)			
CID × MI				−0.078 ** (−2.569)		
CIDNF × MI					−0.057 ** (−2.289)	
CIDF × MI						−0.101 *** (−2.904)
Constant	0.230 *** (17.022)	0.236 *** (16.590)	0.210 *** (21.561)	0.203 *** (25.839)	0.203 *** (25.536)	0.202 *** (27.247)
F	8.941	9.993	3.751	3.386	2.716	3.774
adjusted *R*^2^	0.012	0.014	0.004	0.004	0.003	0.004
VIF	1.014	1.016	1.006	1.005	1.004	1.006

Note: ***, **, *, are 1%, 5%, and 10% significance, respectively.

**Table 4:** Regression results of carbon information disclosure and cost of equity financing.

Variables	(1)	(2)	(3)
CID	−0.076 ** (−2.258)		
CIDNF		−0.070 ** (−2.390)	
CIDF			−0.056 * (−1.919)
FL	0.145 *** (4.259)	0.144 *** (4.237)	0.149 *** (4.382)
OIGR	0.071 *** (2.844)	0.070 *** (2.818)	0.067 *** (2.668)
CS	−0.016 *** (−3.341)	−0.016 *** (−3.409)	−0.018 *** (−3.765)
PID	−0.100 * (−1.713)	−0.099 * (−1.698)	−0.100 * (−1.715)
BM	0.011 (0.667)	0.012 (0.683)	0.010 (0.562)
β	−0.122 *** (−5.753)	−0.122 *** (−5.756)	−0.125 *** (−5.950)
DP	−0.059 *** (−3.124)	−0.059 *** (−3.133)	−0.057 *** (−3.026)
Constant	0.688 *** (6.2063)	0.695 *** (6.330)	0.713 *** (6.526)
adjusted *R*^2^	0.080	0.081	0.078

Note: ***, **, *, are 1%, 5%, and 10% significance, respectively.

**Table 5:** Regression analysis of marketization degree, carbon information disclosure, and cost of equity financing.

Variables	High Marketization Degree (*MI* = 1)			Low Marketization Degree (*MI* = 0)		
	(1)	(2)	(3)	(4)	(5)	(6)
CID				−0.095 ** (−2.026)		
CIDNF					−0.105 ** (−2.588)	
CIDF						−0.091 ** (−2.314)
CID × MI	−0.104 ** (−2.057)					
CIDNF × MI		−0.072 ** (−1.979)				
CIDF × MI			−0.099 ** (−1.985)			
FL	−0.036 (−0.535)	−0.032 (−0.475)	−0.036 (−0.534)	0.102 ** (2.249)	0.098 ** (2.158)	0.107 ** (2.368)
OIGR	0.106 ** (2.189)	0.109 ** (2.228)	0.104 ** (2.147)	0.078 ** (2.373)	0.075 ** (2.280)	0.077 ** (2.298)
CS	0.029 ** (2.466)	0.028 ** (2.330)	0.029 ** (2.425)	−0.015 *** (−2.483)	−0.015 ** (−2.439)	−0.019 *** (−3.109)
PID	0.270 *** (2.665)	0.278 (2.728)	0.257 ** (2.526)	−0.206 *** (−2.606)	−0.206 *** (−2.608)	−0.205 *** (−2.587)
BM	−0.044 (−0.917)	−0.045 (−0.922)	−0.051 (−1.063)	0.011 (0.526)	0.009 (0.446)	0.012 (0.589)
β	0.013 (0.309)	0.013 (0.294)	0.010 (0.239)	−0.151 *** (−5.256)	−0.151 *** (−5.306)	−0.156 *** (−5.418)
DP	−0.034 (−1.427)	−0.035 (−1.430)	−0.031 (−1.309)	−0.067 ** (−2.313)	−0.069 ** (−2.395)	−0.059 ** (−2.058)
F	−0.526 * (−1.878)	−0.512 * (−1.823)	−0.529 * (−1.893)	0.776 *** (5.389)	0.778 *** (5.521)	0.836 *** (5.800)
adjusted *R*^2^	2.262 **	2.126 **	2.295 **	5.834 ***	5.074 ***	4.488 ***
VIF	0.087	0.078	0.089	0.088	0.093	0.080

Note: ***, **, *, are 1%, 5%, and 10% significance, respectively.

**Table 6:** Robustness test results.

Variables	Not Grouped	High Marketization (*MI* =1)	Low Marketizatio (*MI* = 0)
CIDR	−0.076 ** (−2.121)		−0.096 ** (−2.270)
CIDR × MI		−0.071 ** (−2.238)	
FL	0.100 *** (2.675)	−0.047 (−0.708)	0.044 (0.960)
OIGR	0.081 *** (2.604)	0.105 ** (2.172)	0.184 *** (5.550)
CS	−0.013 ** (−2.393)	0.032 *** (2.657)	−0.007 (−1.256)
PID	−0.101 (−1.559)	0.277 *** (2.743)	0.056 (0.703)
BM	0.016 (0.833)	−0.054 (−1.129)	0.010 (0.467)
β	−0.126 *** (−5.091)	0.013 (0.301)	0.031 (1.076)
DP	−0.063 *** (−3.086)	−0.038 (−1.564)	−0.019 (−0.652)
Constant	0.634 *** (5.115)	−0.602 ** (−2.132)	0.296 ** (2.146)
F	4.779 ***	2.398 ***	3.749 ***
adjusted *R*^2^	0.066	0.095	0.064

Note: ***, **, are 1% and 5% significance, respectively.

## References

[1] Development and Reform Commission: Start National Carbon Emission Trading in 2017. http://www.askci.com/news/chanye/2016/01/28/1536268har.shtml.

[2] Jensen M.C., Mechling W.H. (1976). Theory of the Firm: Managerial Behavior, Agency Costs and Ownership Structure. J. Financ. Econ..

[3] Gonzalez-Gonzalez J.M., Ramírez C.Z. (2016). Voluntary Carbon Disclosure by Spanish Companies: An Empirical Analysis. Int. J. Clim. Chang. Strateg. Manag..

[4] Healy P.M., Palepu K.G. (2001). Information Asymmetry, Corporate Disclosure, and the Capital Markets: A Review of the Empirical Disclosure Literature. J. Account. Econ..

[5] Sinkin C., Wright C.J., Burnett R.D. (2008). Eco-efficiency and Firm Value. J. Account. Public Policy.

[6] Dhaliwal D. (2011). Voluntary Non-Financial Disclosure and the Cost of Equity Capital: The Initiation of Corporate Social Responsibility Reporting. Account. Rev..

[7] Zeng Y., Lu Z.F. (2006). The Relationship between Disclosure Quality and Cost of Equity Capital of Listed Companies in China. Econ. Res. J..

[8] Niu S.W., Peng Z.Y. (2016). Study on the Relationship between the Corporate Governance Environment and Internal Control Information Disclosure. Res. Financ. Econ. Issues.

[9] Xu H.P., Lv C.J. (2007). Role of Government, Ownership Proprietary and Cost of Equity. China J. Account. Stud..

[10] Hail L., Leuz C. (2006). International Differences in the Cost of Equity Capital: Do Legal Institutions and Securities Regulation Matter?. J. Account. Res..

[11] The Latest Findings of CDP: Analysis Report on Carbon Information of Chinese Enterprises. http://www.tanjiaoyi.com/article-789-1.html.

[12] Yu H., Tsai B. (2018). Environmental Policy and Sustainable Development: An Empirical Study on Carbon Reduction among Chinese Enterprises. Corp. Soc. Responsib. Environ. Manag..

[13] Guenther E., Guenther T., Schiemann F., Weber G. (2016). Stakeholder Relevance for Reporting: Explanatory Factors of Carbon Disclosure. Bus. Soc..

[14] Kalu J.U., Buang A., Aliagha G.U. (2016). Determinants of Voluntary Carbon Disclosure in the Corporate Real Estate Sector of Malaysia. J. Environ. Manag..

[15] Elijido-Ten E.O. (2017). Does Recognition of Climate Change Related Risks and Opportunities Determine Sustainability Performance?. J. Clean. Prod..

[16] Li D., Huang M., Ren S., Chen X., Ning L. (2018). Environmental Legitimacy, Green Innovation, and Corporate Carbon Disclosure: Evidence from CDP China 100. J. Bus. Ethics.

[17] Kolk A., Levy D., Pinkse J. (2008). Corporate Responses in an Emerging Climate Regime: The Institutionalization and Commensuration of Carbon Disclosure. Eur. Account. Rev..

[18] Li S., Zhao Y., Tong J. (2013). Can Corporate Social Responsibility Report Reduce Cost of Equity Capital?: Evidence from Chinese Stock Market. China J. Account. Stud..

[19] Kim E.H., Lyon T. (2011). When Does Institutional Investor Activism Increase Shareholder Value?: The Carbon Disclosure Project. BE J. Econ. Anal. Policy.

[20] Lu W.B., Guan F., Zhang P.P., Deng Y.J. (2014). Media Coverage, Information Disclosure Environment and Equity Cost. China J. Account. Stud..

[21] Diamond D.W., Verrecchia R.E. (1991). Disclosure, Liquidity, and the Cost of Capital. J. Financ..

[22] He Y., Tang Q., Wang K. (2013). Carbon Disclosure, Carbon Performance, and Cost of Capital. China J. Account. Stud..

[23] Ren Y.J., Qiao W.H. (2016). Study on the Relationship between the Environmental Accounting Information Disclosure Quality and Cost of Financing. J. Dalian Marit. Univ. (Soc. Sci. Ed.).

[24] Ye C.G., Wang Z., Wu J.F., Li H. (2015). External Governance, Environmental Information Disclosure and the Cost of Equity Financing. Nankai Bus. Rev. Int..

[25] Zhang J., Wang J.C., Gong X.L. (2015). Carbon Information Voluntary Disclosure and the Equity Capital Cost. Mod. Manag. Sci..

[26] Yuan Y. (2014). Environmental Information Disclosure Quality and Cost of Equity Financing: The Empirical Evidence of Heavy Pollution Industry from Shanghai A-share. J. Zhongnan Univ. Econ. Law.

[27] Ye K.T., Lu Z.F. (2004). China’s Listed Company Equity Financing Costs Influence Factors Analysis. Manag. World (Mon.).

[28] Herold D. (2018). Has Carbon Disclosure Become More Transparent in the Global Logistics Industry? An Investigation of Corporate Carbon Disclosure Strategies between 2010 and 2015. Logistics.

[29] Qian W., Hörisch J., Schaltegger S. (2018). Environmental Management Accounting and Its Effects on Carbon Management and Disclosure Quality. J. Clean. Prod..

[30] Lee S.Y., Park Y.S., Klassen R.D. (2015). Market responses to firms’ voluntary climate change information disclosure and carbon communication. Corp. Soc. Responsib. Environ. Manag..

[31] Herold D.M., Lee K.H. (2018). The Influence of Internal and External Pressures on Carbon Management Practices and Disclosure Strategies. Australas. J. Environ. Manag..

[32] Luo L., Tang Q., Lan Y.C. (2013). Comparison of Propensity for Carbon Disclosure between Developing and Developed Countries: A Resource Constraint Perspective. Account. Res. J..

[33] Kang J.J., Wang W., Fu Y.Y. (2009). Empirical Analysis on Spatial Linkages in Marketization of China’s Provinces. Stat. Res..

[34] Fan G., Wang X.L., Ma G.R. (2011). Contribution of Marketization to China’s Economic Growth. Econ. Res. J..

[35] Sun Z., Liu F.W., Li Z.Q. (2005). Market Development, Government Influence and Corporate Debt Maturity Structure. Econ. Res. J..

[36] Chen W.T., Li X.C. (2008). Ownership Concentration, Risk Propensity and Market Value of Listed Family Firms: An Empirical Study Based on Market Development Degree Grouping. China Ind. Econ..

[37] Cheng X.S., Tan Y.C., Xu L. (2011). The Company Value, Voluntary Disclosure and Marketization Process: Based on the Qualitative Information Disclosure. J. Financ. Res..

[38] Chen X.L., Lin X. (2010). Marketization, Big Shareholders Occupy and Information Transparency: The Evidence from China’s Securities Market. J. Financ. Econ..

[39] Cai H.J., Xu H. (2016). Process of Marketization, Investor Attention and Investment Efficiency. Collect. Essays Financ. Econ..

[40] Cui X.M., Liu J. (2009). The Process of Marketization, the Nature of Ultimate Ownership and Corporate Social Responsibility: Empirical Evidence from Shanghai Securities Exchange. Soft Sci..

[41] Wang Y.M., Liu W.B., Liu Y.H. (2015). Walking the Talk? What Firm Say about CSR Verse What They Do with CSR. China Soft Sci..

[42] Lian J. (2012). Political Connection, Marketization Process and the Cost of Equity Capital: Empirical Evidence from Chinese Private Listed Companies. Res. Econ. Manag..

[43] Du X.Y., Ma R.G. (2016). Explore and Analyze on the Relationship of Marketization Degree, Institution Arrangement and Anti-corruption: Based on the Macro Panel Data of 2000-2013. Stat. Inf. Forum.

[44] Li L., Liu Q.Q., Tang D.L., Xiong J.C. (2017). Media Reporting, Carbon Information Disclosure, and the Cost of Equity Financing: Evidence from China. Environ. Sci. Pollut. Res..

[45] Li H.Y., Liu D. (2016). Marketization Process, Voluntary Disclosure and the Cost of Equity Capital. China J. Account. Stud..

[46] Shen H.T., Feng J. (2012). Media Monitoring, Government Supervision, and Corporate Environmental Disclosure. China J. Account. Stud..

